# PlasmaDNA: a free, cross-platform plasmid manipulation program for molecular biology laboratories

**DOI:** 10.1186/1471-2199-8-77

**Published:** 2007-09-17

**Authors:** Alexandre Angers-Loustau, Jeffrey Rainy, Kirmo Wartiovaara

**Affiliations:** 1 Institute of Biomedicine, University of Helsinki, Helsinki, Finland; 2 Département d'Informatique et de Recherche Opérationnelle, Université de Montréal, Canada

## Abstract

**Background:**

Most molecular biology experiments, and the techniques associated with this field of study, involve a great deal of engineering in the form of molecular cloning. Like all forms of engineering, perfect information about the starting material is crucial for successful completion of design and strategies.

**Results:**

We have generated a program that allows complete *in silico *simulation of the cloning experiment. Starting with a primary DNA sequence, PlasmaDNA looks for restriction sites, open reading frames, primer annealing sequences, and various common domains. The databases are easily expandable by the user to fit his most common cloning needs. PlasmaDNA can manage and graphically represent multiple sequences at the same time, and keeps in memory the overhangs at the end of the sequences if any. This means that it is possible to virtually digest fragments, to add the digestion products to the project, and to ligate together fragments with compatible ends to generate the new sequences. Polymerase Chain Reaction (PCR) fragments can also be virtually generated using the primer database, automatically adding to the fragments any 5' extra sequences present in the primers.

**Conclusion:**

PlasmaDNA is a program available both on Windows and Apple operating systems, designed to facilitate molecular cloning experiments by building a visual map of the DNA. It then allows the complete planning and simulation of the cloning experiment. It also automatically updates the new sequences generated in the process, which is an important help in practice. The capacity to maintain multiple sequences in the same file can also be used to archive the various steps and strategies involved in the cloning of each construct. The program is freely available for download without charge or restriction.

## Background

Numerous programs and web servers have emerged to analyze primary DNA sequence for recognition sites of restriction enzymes, the primary tools used in DNA cloning experiments [1-6]. Another primary sequence analysis often included is the recognition of open reading frames (ORFs) using the genetic code as a guide. These programs provide a visual, graphical representation of the DNA sequences. Some of them, like PlasMapper, provide additional information by analyzing the DNA for functional domains using a database of common plasmid features [1].

Although these programs provide excellent information about a particular sequence, which is usually the starting material of the experiment, their weakness reside in generating new sequences to keep track of the DNA sequences produced by successful cloning. This usually leads to hazardous copy/paste manipulations between multiple sequence files, or to complete lack of sequences for new plasmid constructs, undermining the successful use of the DNA constructs for future cloning processes.

PlasmaDNA has been designed to incorporate the existing elements which are most useful for virtual cloning in a simple, free to use software. The most important elements include the capacity to manipulate more than one sequence in the same session and save them in the same file, as well as adding the additional information of "overhangs" to the primary DNA sequence. Identical versions of the program exist for both Windows [see Additional file 1] and Apple [see Additional file 2], with compatible save files, such that most platform issues between laboratories can be eliminated.

## Implementation

PlasmaDNA is written in C++, and consists of two elements: a back-end "Cloning Project" containing the DNA sequences (up to 20 per session) and the databases of restriction enzyme recognition sites, primers and common domain that are used to analyze them, as well as a front-end user interface generated using the Jules Utility Class Extensions [7]. This toolkit, for which the authors hold a single-program commercial license, allows identical, full functionality of PlasmaDNA on Windows and Apple operating systems.

## Results

### Primary sequence analysis

DNA sequences can be added one by one to the project, using simple copy/paste in the appropriate window, or in bulk through compatibility with multi-sequence FASTA files generated, for example, by GeneBank.

The primary sequence of each fragment is analyzed for the presence of multiple shorter sequences. First, restriction sites are marked and displayed on the graphical view. Second, primer annealing sequences are identified, which can be used to generate new fragments by a virtual simulation of polymerase chain reaction (PCR, see below). Third, open reading frames are identified using simple genetic code. The cut-off for protein length has been set up to 150 codons between the ATG and a STOP codon. Finally, the sequence is analyzed for the presence of common domains found in current commercially available plasmids, from a database included with the program.

All the analyses are performed using simple text matching, and a domain will only be identified if it is present in the sequence with 100% homology, and from the first to the last base. This allows the program to identify and differentiate point mutants of the same sequence, and to show the user whether the domain remains intact and whole through the various manipulations.

**Figure 1:**
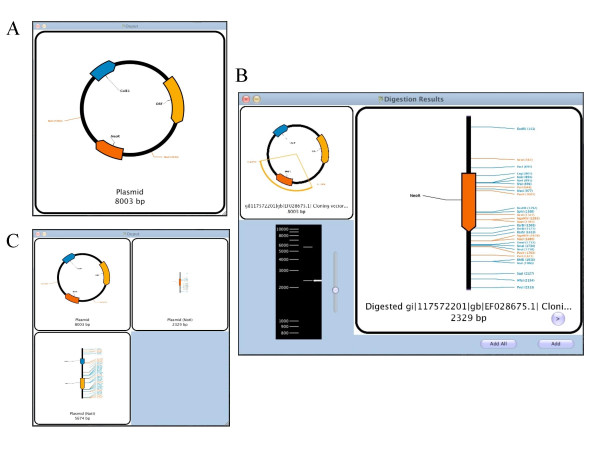
**Virtual restriction digest of a plasmid**. A) Output Window of a project containing a single plasmid with two shown NotI sites. B) Digestion window selecting NotI as an enzyme. C) After adding both digestion products to the project, the project contains three fragments, including the original plasmid. Each of the new fragments can then be further analyzed and manipulated. The fragments can be visualized together and to scale, as shown, or one by one for greater resolution.

The result of these analyses is shown in a graphical format in the output window (Figure 1). The user can chose between restriction, primer or ORF analysis to be overlaid to the domains view, as well a graphical representation of the %GC in small stretches along the primary DNA sequence. The output is color-coded, using a palette of colors that is unambiguous even for people with different kinds of color blindness [8]. The output can be exported as a graphical file of the jpg format for inclusion in various other documents or presentations.

### Databases

The three main primary sequence-matching analyses performed by PlasmaDNA on DNA sequences involve restriction sites, domains and primers. The internal databases are built from sequences read from three database files, Enzymes.pdat, Domains.pdat and Primers.pdat, respectively, when the program is started.

The restriction enzyme database supplied with the program contains 75 enzymes recognizing sequences which are non-degenerate and at least 6 bases long, since these are the enzymes most often used for cloning experiments. The domains database contains 162 sequences representing various elements commonly found in commercial vectors such as antibiotic resistance genes and fusion tags (see Table 1). The primers database, in the downloaded package, contains no element.

**Table 1:** Summary of the different domains included in the release version of the database

Type	Number
Resistance genes	28
Fusion tags	17
Other coding sequences	39
Origins of replication	13
Promoters	24
Sequencing primers	7
Recombination sites	10
PolyA signals	9
Others	15
Total	162

All these databases can be easily expanded to match the needs of the individual users. Adding domains and primers sequences is performed using the appropriate user interface elements in PlasmaDNA, as explained in the included manual. For domains, the new sequence can be added to the Domains.pdat file, in which case all further sessions of PlasmaDNA will include it in the analyses, or to the current project, in which case the domain will be saved in the project save file and included only when the project is loaded.

Other database manipulations, such as expanding the enzymes database, as well as editing or deleting elements found in the three databases, can be achieved using an included application called PlasmaDNA Database Manager (PlasmaDMan). This application uses a simple interface to allow the user to manipulate the databases at will. Elements of the databases can be deleted permanently, or temporarily transferred to other sub-files that are not automatically loaded by the main PlasmaDNA application, but can be kept for future access when needed. Instructions for use of this application are included in the program manual.

### Virtual Cloning

As described before, PlasmaDNA can contain multiple sequences in one session, and keeps track of the fragments' overhangs. This means that it is possible, using the program, to digest a fragment to obtain the digestion products, and to ligate compatible fragments to obtain the ligation products.

Digestion of a fragment is performed by clicking the "Digest" button of the corresponding fragment and by choosing which enzyme(s) to add to the digestion mix. The resulting fragments are shown in a scrollable manner, and their positions on the original fragment and on a virtual agarose gel are highlighted (Figure 1B). The desired digestion products can be added to the project, with attached overhangs corresponding to the ones generated by the enzymes (Figure 1C).

**Figure 2:**
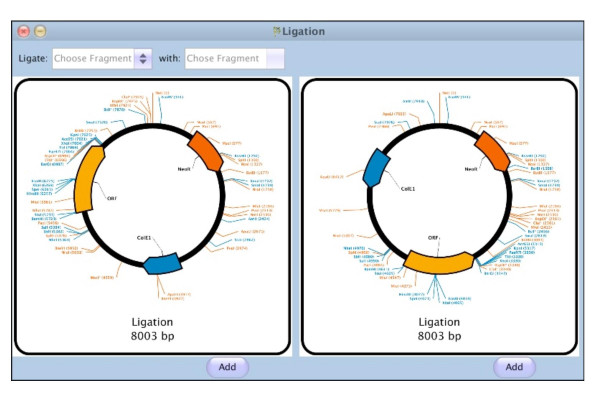
**Virtual ligation of the digestion fragments from Figure 1**. The two possible ligation products are generated and shown, and the user can decide to add one or both to the current project.

Ligation is performed by selecting compatible fragments in a "Ligation" sub-window. The first fragment is selected from the list of all non-circular fragments. The second fragment is then selected from a list containing only compatible fragments, i.e. fragments which have at least one overhang compatible with the first fragment. The Ligation window then shows the possible ligation product(s), with the possibility to add them one by one to the project (Figure 2).

### Polymerase Chain Reaction

**Figure 3:**
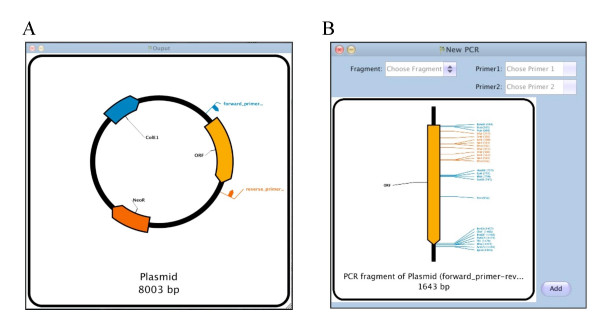
**Virtual PCR**. A) Plasmid from Figure 1A, with the output options set on "Primers Analysis" showing two primers binding sites in an orientation favorable for a PCR. B) PCR window, selecting Plasmid X as the template, and the two primers. The resulting fragment can be added to the project to be further analyzed and manipulated.

Another molecular biology method commonly used to generate new fragments is the Polymerase Chain Reaction (PCR). PlasmaDNA can also simulate this technique, by analyzing the sequence for annealing sites of user-defined primers. These primers can contain extra non-annealing sequences in their 5' end, such as those used to add restriction sites at the ends of PCR products. Using the PCR sub-window, the user can select a template fragment, a sense and an antisense primer. The product is generated, adding the corresponding extra 5' sequences on both ends if present, and can be added to the project for further manipulations (Figure 3).

### Saving and exporting

Every text field from PlasmaDNA is compatible with the clipboard and copy/paste functions, and the graphical element of individual fragments can be exported as graphic files of the JPEG format.

All the sequences in the same project are saved by PlasmaDNA in the same file, which will contain the primary sequence of each fragment, their overhangs, whether or not they are circular, as well as user-defined preferences for showing/not showing different enzymes, domains, and primers. In addition, if some domains were added to this "project only", to only analyze fragments in this project for their presence, these domains will be added at the end of the save file, to be re-loaded when the project is opened.

PlasmaDNA allows the presence of multiple fragments in the same file. This feature can used for various useful purposes: for example, it is possible to keep, together with the new construct, the original plasmids from which the pieces were taken, as well as the various digested fragments that were ligated together. This allows a sure and convenient way to store the cloning strategy for future reference. Also, it is possible to group various related plasmids (for example, different multiple cloning sites phases for the same fusion tag) in the same file, allowing laboratories to group and organize their plasmid collection for convenient access when a certain plasmid is needed in a cloning strategy design.

When the user loads a file containing multiple sequences, a window will appear to show a preview of the name and graphical map of the fragments present in the file. It is then possible to choose which of the fragment(s) to load and add to the current project.

It is also possible to export the primary sequences in the FASTA format. Although this will eliminate some information such as overhangs, this format is compatible with most existing DNA analysis programs.

## Discussion

Although not unique in its capacity to analyze primary DNA sequences, PlasmaDNA focuses on what these analyzes are mostly needed for: planning a practical molecular biology laboratory cloning experiment and generating the resulting sequences.

By making this simulation visual and simple, it allows a full preparation of a cloning experiment, from beginning to end, before starting the actual manipulations. This can save time and money for cloning projects as it minimizes the chances of having to restart halfway through due to an unnoticed, undesirable restriction site or improper calculation of the phase for fusion proteins. The program can also be used in academic settings to teach students about the concepts of cloning and providing them with a visual representation of what happens in the different steps.

Finally, PlasmaDNA makes it easy and error-proof to obtain the new sequences generated during cloning processes. This will ensure that this new generation of plasmids and constructs can be used for further cloning experiments with complete maps and restriction analysis, once again facilitating the process and preventing waste. The program itself relies on a simple, intuitive, entirely button-based interface. An instruction manual is included in the downloadable package found on the website.

## Availability and requirements

**Project name**: PlasmaDNA

**Project home page**: 

**Operating system(s)**: Windows and MAC

**Programming language**: C++

**Other requirements**: None

**License**: Freeware

**Any restrictions to use by non-academics**: None

## Competing interests

The author(s) declares that there are no competing interests.

## Authors' contributions

The structure and implementation of the program was the work of AA-L, with assistance from JR. KW supervised the project and contributed to the drafting of the manuscript. All authors have read and approved the final manuscript.

## Supplementary Material

Additional file 1 PlasmaDNA Windows version package. Version 1.3.6 of the PlasmaDNA package for Windows Click here for file

Additional file 2 PlasmaDNA MacOSX version package. Version 1.3.6 of the PlasmaDNA package for MacOSX Click here for file
