# Supplementary material for: PlasmaDNA: a free, cross-platform plasmid manipulation program for molecular biology laboratories
Source: BMC Mol Biol. 2007 Sep 17;8:77. doi: 10.1186/1471-2199-8-77 (PMC2075515; doi:10.1186/1471-2199-8-77)
Supplement: Additional file 1 — PlasmaDNA Windows version package. Version 1.3.6 of the PlasmaDNA package for Windows [file 1471-2199-8-77-S1.zip › PlasmaDNA/PlasmaDNA manual.pdf]

PlasmaDNA:

a PLASmid MAnipulation program

2007, by Alexandre Angers-Loustau

Introduction

About the Program

Plasma DNA is a free, cross-platform DNA manipulation program started in the end of 2006 by Dr Alexandre Angers-Loustau, a molecular biologist, while performing post-doctoral studies at the University of Helsinki.

The aim of the program is to allow analysis and manipulation of DNA sequences in a simple and intuitive manner, targeting the everyday needs of scientists doing a lot of cloning and helping them keep track of the new sequences they produce.

PlasmaDNA features:

- Full functionality on both Windows and MacOS operating systems, including compatibility of the saved files.

- Automatic analysis of DNA sequences for restriction sites, primer binding sites, and common plasmid domains from easily-expandable (included) databases.

- Capacity to keep more than one sequence in the same file. Since PlasmaDNA keeps track of the overhangs of each fragment, this allows:

- *In silico* digestion with restriction enzymes, and ligation of compatible fragments to automatically generate the new sequences produced by real-life cloning.

- *In silico* Polymerase-Chain-Reaction (PCR).

- *In silico* GATEWAY recombination between compatible fragments.

- Multiple sequence Import/Export compatibility with the FASTA format.

License

PlasmaDNA is a freeware, i.e. when you start it, you automatically agree to the following license:

You are can use PlasmaDNA on as many computer computers as you like, for as long as you like, without charge. You are allowed, and encouraged, to distribute the program, provided that you distribute it as a complete package. This includes:

-PlasmaDNA.exe (Windows) or PlasmaDNA.app (MacOS)
-This manual, including this license
-Enzymes.pdat
-Domains.pdat
-Primers.pdat
-PlasmaDNA logo.png (Windows only)

It is highly recommended to download a copy directly from the author by going to the website http://research.med.helsinki.fi/plasmadna/.

This program is provided by the author with the hope that it can be as useful to you as it is to himself. The author makes no guarantee, express or implied, of merchantability and fitness for a particular purpose, and can not be held responsible for direct or indirect damage, waste of material or wrong information resulting from the use of this program.

For feedback, questions, comments, or to report bugs, feel free to write me at http://research.med.helsinki.fi/plasmadna/Feedback/Feedback.htm/.

Using PlasmaDNA

Main Window

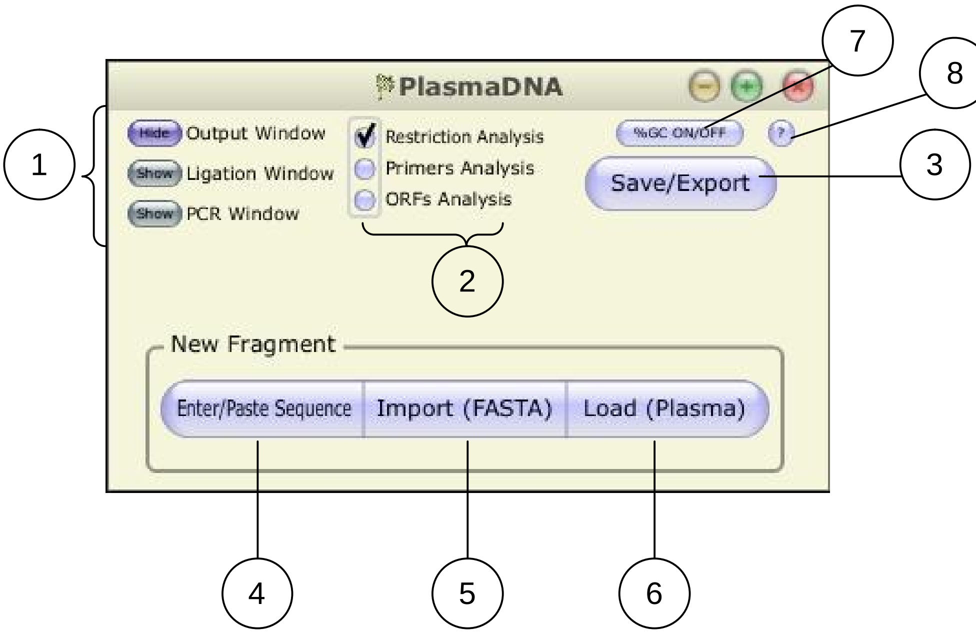

1: Secondary windows show / hide.

2: Output window content selection.

3: Save / Export window.

4: Add a new fragment by typing / pasting the sequence.

5: Add (a) new fragment(s) by importing from a FASTA-format file.

6: Load a file previously saved by PlasmaDNA.

NOTE: the fragments in the loaded files will be ADDED to the fragments already present in the PlasmaDNA session.

7: Show/Hide the overlay of percent GC content of the DNA fragment.

8: Opens the "About" window to see the current version number.

From this menu, you can add new fragments (through copy/paste, importing or loading) to the current session, show / hide the different secondary windows that allow you to visually analyse the fragment(s), perform PCR or ligate compatible fragments.

The previous figure showed the default state of the main window, without any fragment loaded. As you add new fragments, the following menu will appear, one for each fragment:

Fragment Menu

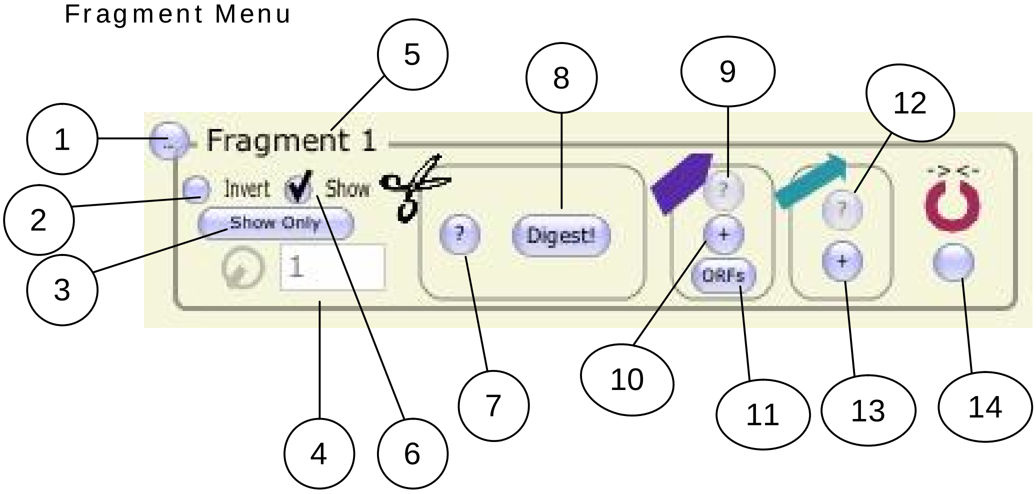

1: Edit / View Sequence window.
2: Invert DNA fragment (reverse-complement).
3: Show only button: will show this fragment, and hide all the others, in the Ouput window.
4: Rotate Fragment : enter the base that should become the new 1 position. This is only available for circular fragments.
5: Name of the Fragment.
6: Show / Hide fragment in the Output window.

7: Restriction Enzyme info window for this fragment.
8: Digest this fragment with a restriction enzyme. This will open the enzyme selection window.

9: Domains info window for this fragment.
10: Add a domain from this fragment.
11: Show Open Reading Frames and translations for this fragment.

13: Primers info window for this fragment.
14: Add a new primer to the PlasmaDNA database.

15: Circularize this fragment (in case the automatic analysis failed to recognize and circularize a circular DNA fragment)

One of this menu will appear on the main window for each of the fragments loaded on the current session. The limit has been set at 20 fragments at the same time. From this menu, you can manipulate the fragments (inversion, rotation, digestion), and modify the look of the fragment in the output window through the various sub-windows.

Fragment sub-windows

Edit/View Sequence:

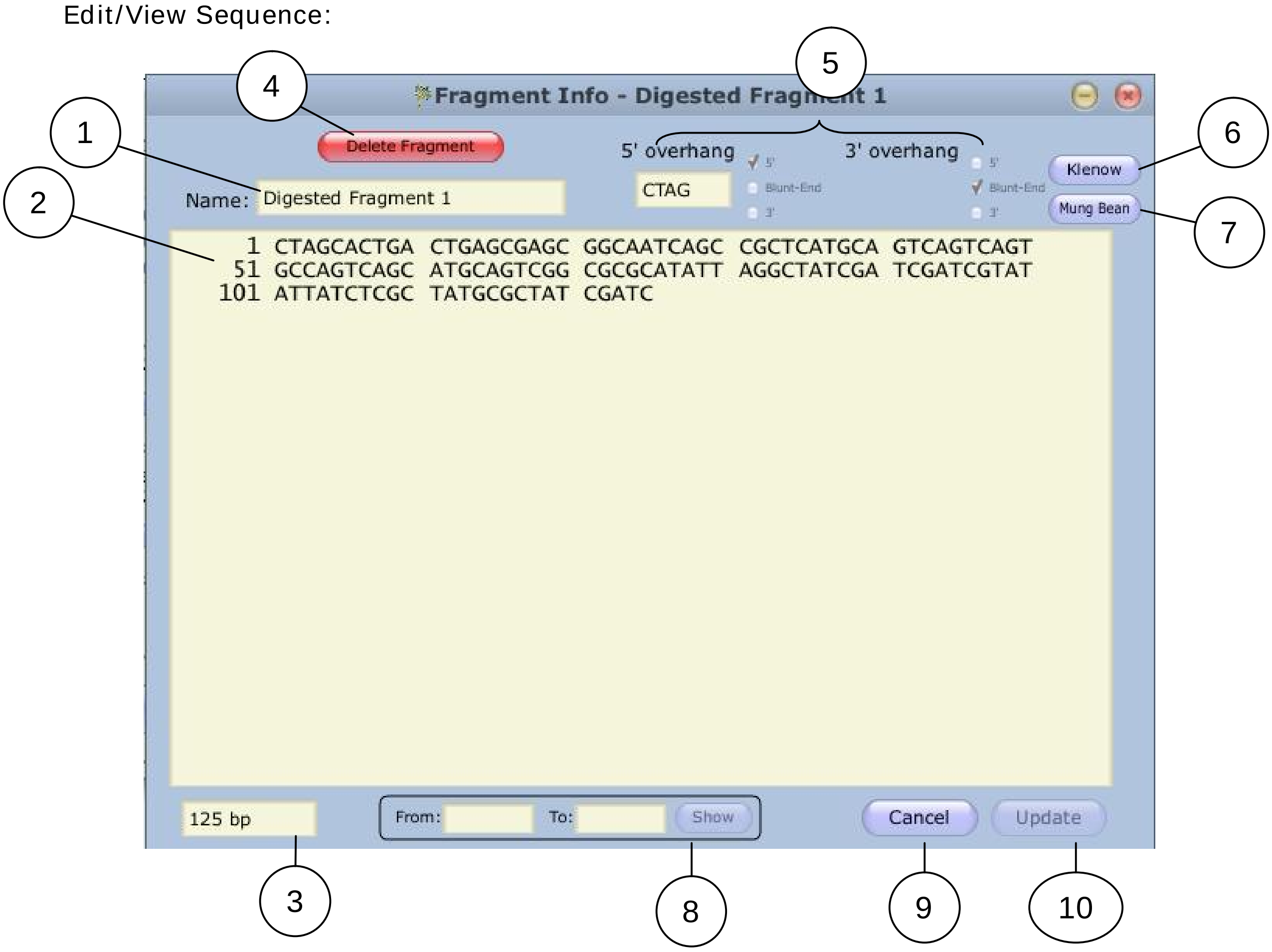

1: View / Edit name of the Fragment
2: View / Edit sequence of the Fragment
3: Size of the fragment in base pairs (note: this field is not editable)

4: Delete Fragment (Warning! This is not reversible)

5: 5 ’and 3 ’overhangs of the fragment. (note: these fields are not editable unless you are entering a new fragment)

6: Blunting of the fragment using the Klenow polymerase-type rules.
7: Blunting of the fragment using Mung-Bean nuclease-type rules.
Warning: these last two are not reversible.

8: Select and show a sub-sequence of the current fragment.

9: Exit without saving the new name and/or sequence.
10: Save the new name and/or sequence and exit.

Clarification of the nomenclature:

The fragment:

AATCGGCGTA
     CCGCATTA

Has a 5 ’overhang which is of type 5 ’and of sequence AATC
Has a 3 ’overhang which is of type 5 ’and of sequence AT
The corresponding shown sequence would be AATCGGCGTA
After inversion, the shown sequence would be ATTACGCC

Select Enzyme:

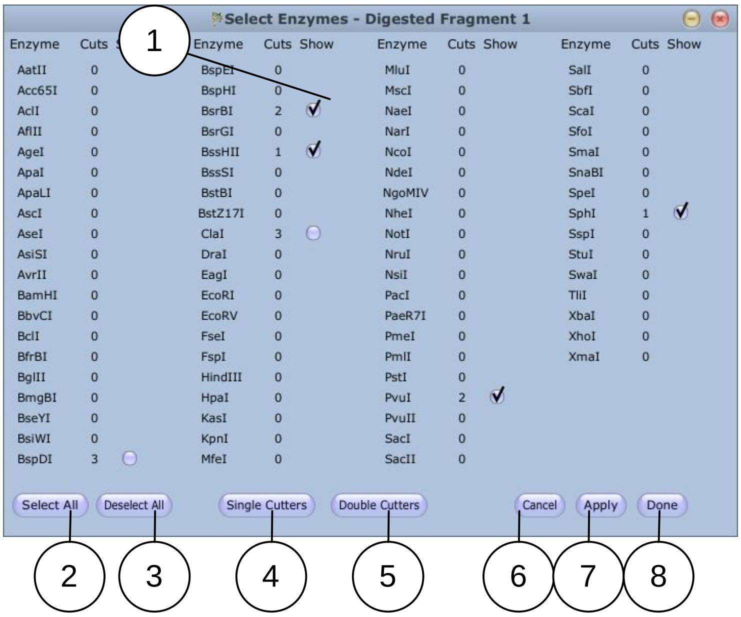

1: Click to show / hide this enzyme: If the enzyme is selected, it will be shown for this fragment in the Output Window.

2: Select all enzymes.
3: Deselect all enzymes.

4: Select only the single cutters.
5: Select only the enzymes cutting twice.

6: Cancel - Close the window without applying the changes.
7: Apply – Apply the changes without closing the window.
8: Done – Apply the changes and close the window.

This window is used to select which enzymes which enzymes will be shown on the Ouput Window (if the output type is set to ‘Restriction Analysis’, see page 4)

This window is NOT used to digest the fragment. For this, use the ‘Digest!’ button in the fragment menu (number 8, page 5). A similar window will open where the desired enzyme(s) can be chosen. Clicking ‘Digest’ in this sub-window will open the following Digestion Window:

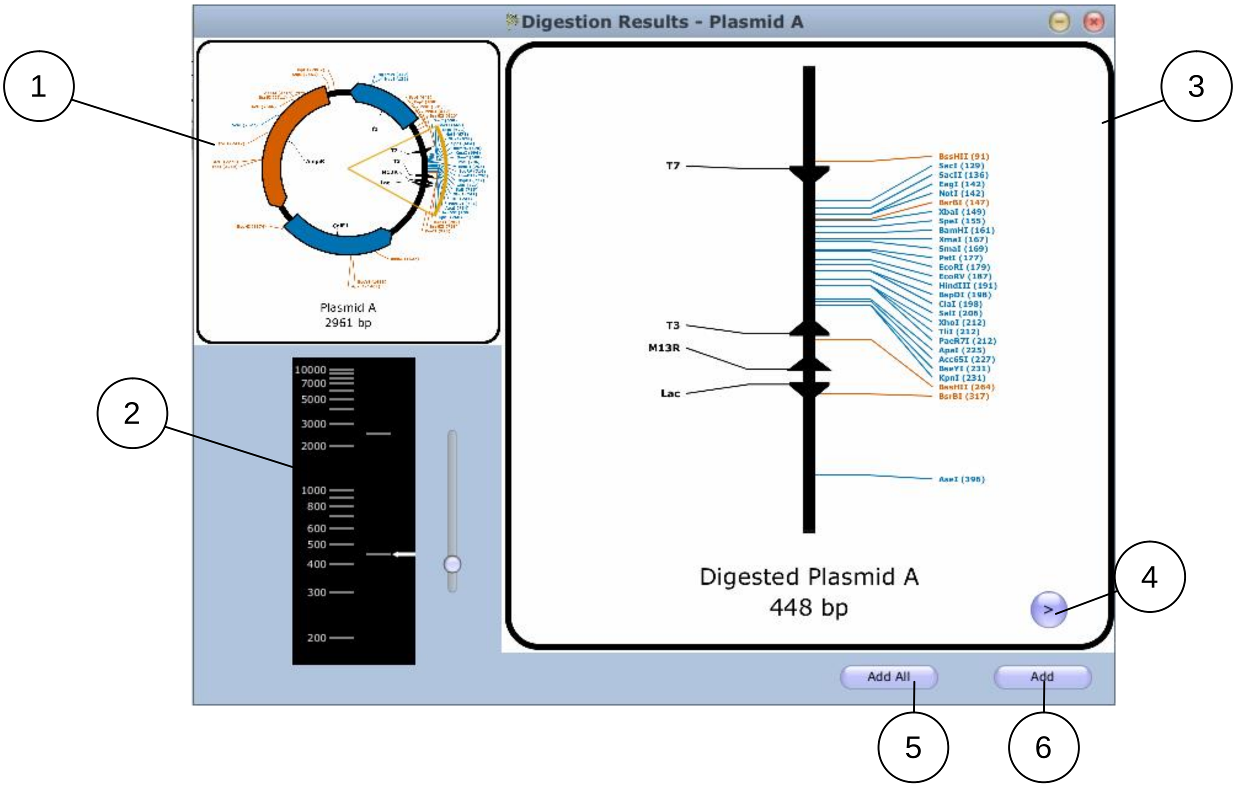

1: Graphical view of the substrate fragment. The orange line represents the region of the original fragment corresponding to the shown digestion fragment.

2: Virtual Gel showing the expected bands as they would migrate compared to a 100bp/1kb ladder. The shown digestion fragment is identified with an arrow. The percent agarose of the gel can be changed using the slider on the right of the gel.

3: Digestion fragment.
4: Show next digestion fragment.

5: Add all digestion fragments to the current project.
6: Add the shown digestion fragment to the current project.

Select Domains:

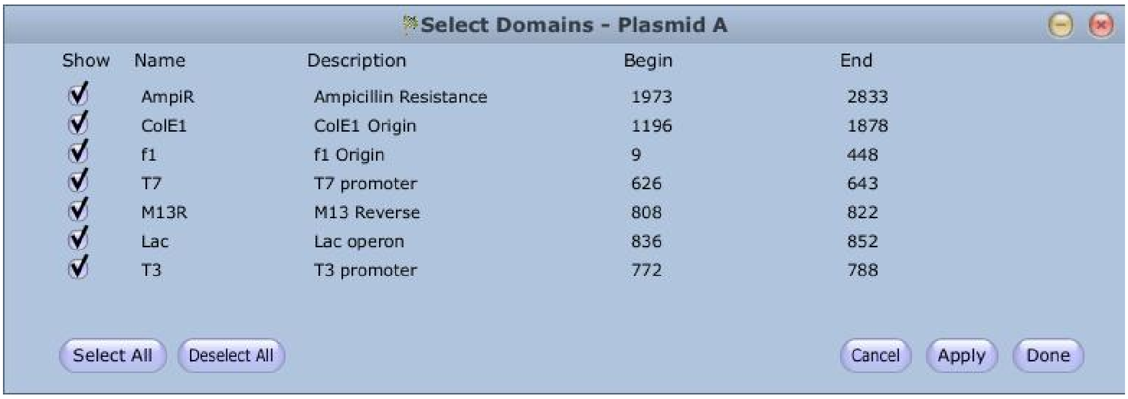

This window is used to select which domains will be shown in the Ouput Window for this fragment. The buttons are similar in function to the Select Enzymes window (see page 7). Besides the name of the domain as shown on the Output Window, additional informations include a short description of the domain, as well as the begin and end base number. If a domain is known to exist on the fragment, but was not detected by the program, you can add it using the ‘add Domain ’button (number 10 from page 5). The following window will appear:

Add Domain:

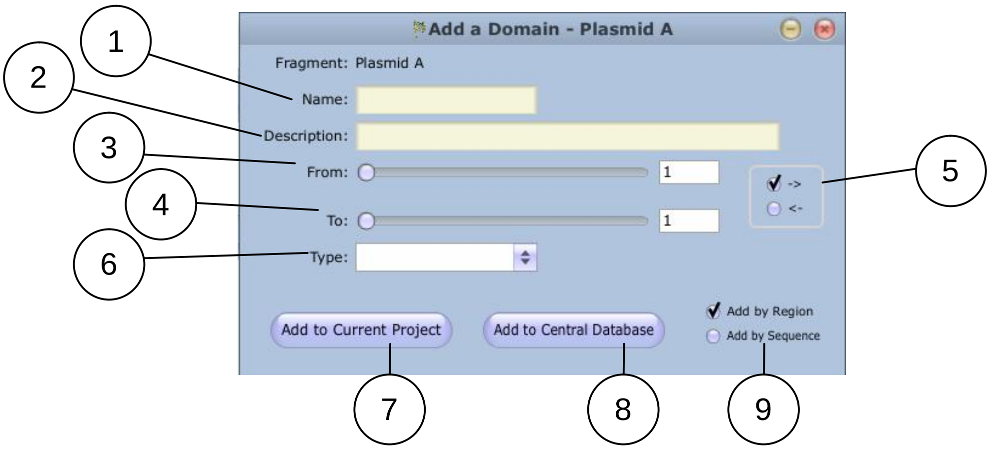

1: Short name for the domain.
2: Description for the domain.
3: Base number for the beginning of the domain.
4: Base number for the end of the domain.
5: Orientation of the new domain (for circular fragments only – see below).
6: Type of sequence represented by this domain. This will determine the color used by the program to draw the domain.

7: Add the domain to a local database, i.e. only the fragments (present and future) from the current file will be analyzed for the presence of this domain
8: Add the domain to the file Domains.pdat, which means that all the fragments will be analyzed for the presence of this domain

Note: You can only add the domain once ALL the fields have been filled. The addition has been succesful once the window has closed.

9: Add by Region or Add by Sequence (see below).

Orientation of the new domain for “Add by Region”:

For linear fragments, to add a domain on the sense strand, enter a from ’ base SMALLER than a to ’base. To add a domain on the antisense strand (reverse orientation), enter a from ’base BIGGER than a to ’base.

For circular fragments, follow the following rules:

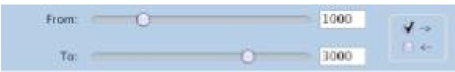

Will result in:
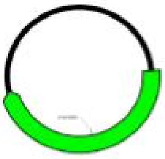

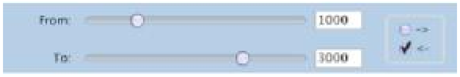

Will result in:
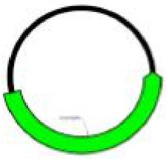

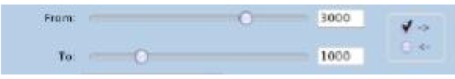

Will result in:
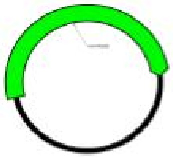

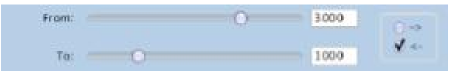

Will result in:
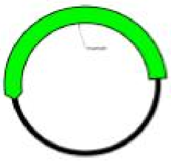

Alternatively, if you know the exact primary sequence of the domain you wish to add, you can select "Add by sequence" (button 9). The window will then look like this:

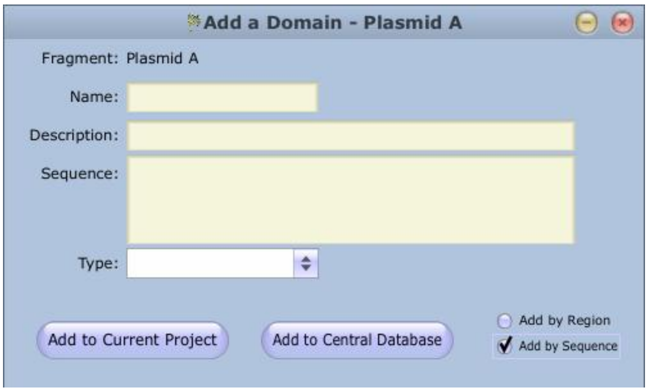

View Open Reading Frames:

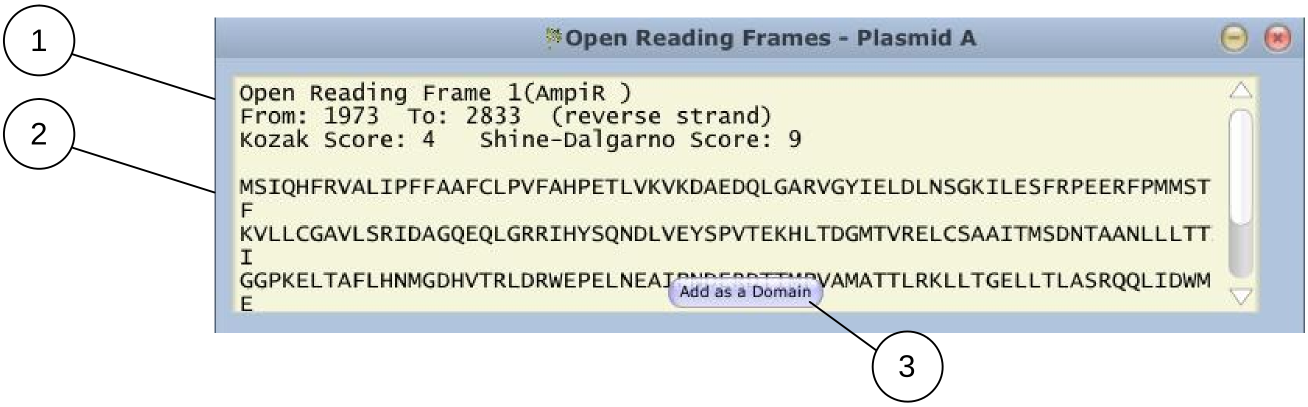

1: ORF information, including the number/name it appears under in the Output Window (when the 'ORF Analysis' is selected), the begin/end bases, and the arbitrary Kozak and Shine-Dalgarno scores calculated from the primary DNA sequence.

2: Translation of the ORF, using the standard mammalian genetic code.

3: Add the current ORF as a domain. This will open an Add Domain window, with fields 3, 4 and 5 pre-filled (see page 9) to correspond to this ORF.

Select Primers:

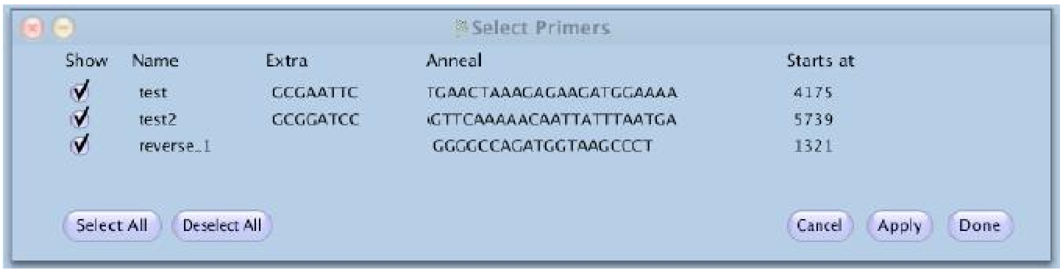

This window is used to select which primers will be shown in the Ouput Window for this fragment. The buttons are similar in function to the Select Enzymes window (see page 7). Besides the name of the primer additional information includes the annealing sequence of the primer, the extra (non-annealing) 5 ' sequence present in this primer, as well as the base at which it starts to anneal on the fragment. To add a primer to the database, use the Add Primer function of the main Fragment menu (number 13 from page 5).

Add Primer:

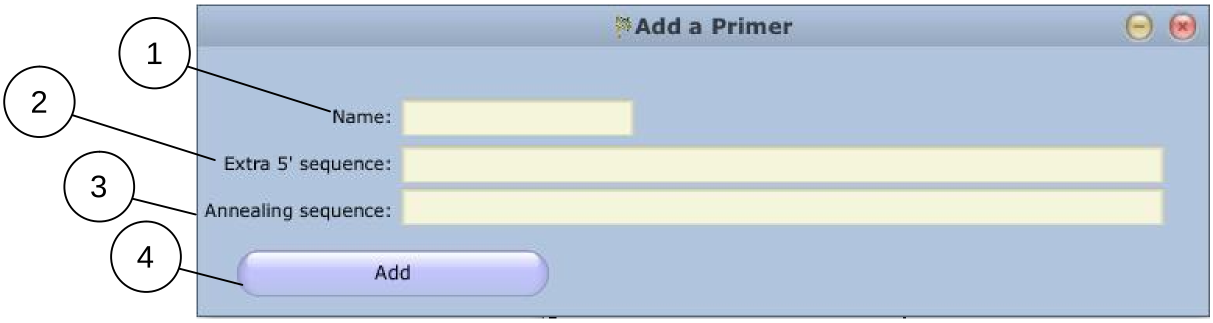

1: Name of the primer. This field should be without spaces, any space present will be replaced with underscores ("_ ").

2: Extra 5 'sequence that does not anneal to the template DNA. For example, when a restriction site is added to the end of the PCR product.

3: Sequence from the primer that anneals to the template. This sequence will be used by the program to identify primer binding sites. Only non-degenerate sequences are accepted for now.

4: Add button, this will add the primer to the Primers.pdat files and re-analyze the current fragments for annealing sites.

Note: To cancel, just close the window (top-right red button).

How to perform a ligation

Since PlasmaDNA can contain more than once fragment in the same session, and since it keeps track of the overhangs present at the ends of the fragments, it is possible to 'ligate' compatible fragments in a way that mimics the events of a cloning experiment. PlasmaDNA automatically generates the resulting products, which minimizes the mistakes possible with the current copy/paste strategies.

Ligations are performed using the Ligation Window (see 1 on page 4). For example, if a project has 2 fragments, both having XhoI overhangs at both ends.

1) Open the Ligation Window and select one of the fragments in the 'Ligate: 'box

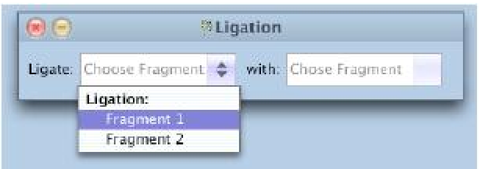

2) Select the second fragment from the 'with: ' box. Note: only fragments compatible with the first fragment will appear as choices in this box.

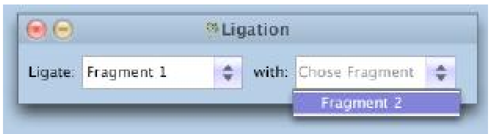

3) The possible ligation products will then appear in the Ligation Window:

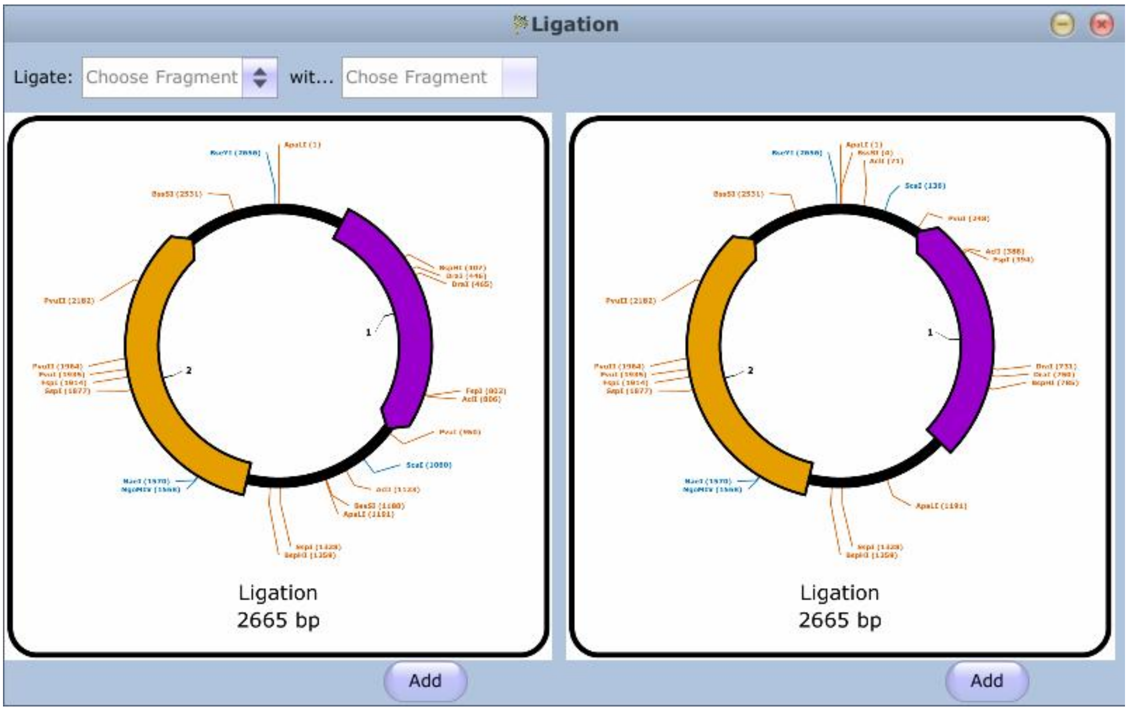

From there, you can select which one(s), if any, you want to add to your current project as a new fragment by clicking on the Add ' button under the corresponding image.

Note: To perform Gateway recombinations between compatible fragments, just select the two fragments as Ligate: ' and with: ' choices. The resulting recombinations products will appear in the Ligation window.

How to perform a PCR

If, on the same fragment, you have two primers annealing sites that face each other, you can use PlasmaDNA to generate the product of the resulting PCR and add it to your project. For this, use the PCR Window (see 1 from page 4)

For the following Fragment:

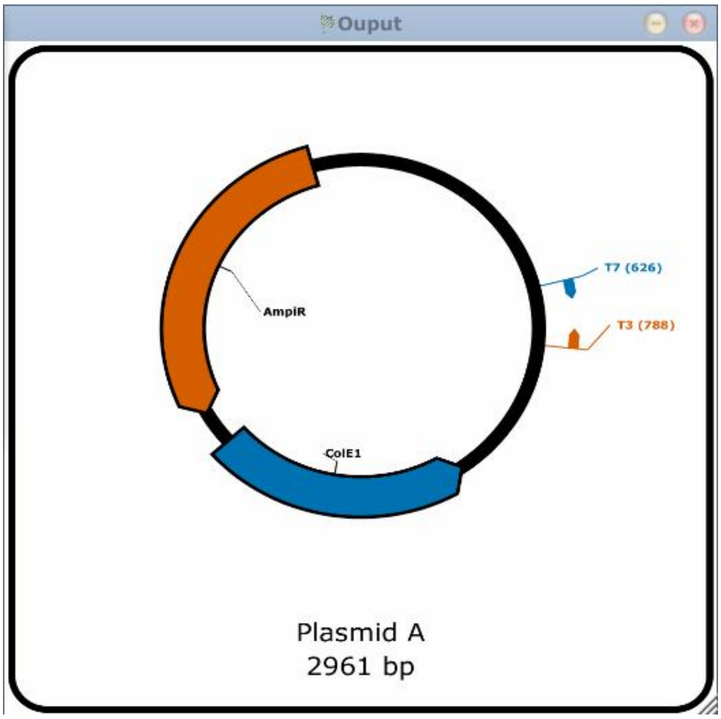

The primers T7 'and T3 'can be used for a PCR. First, open the PCR Window and select the fragment to use as template:

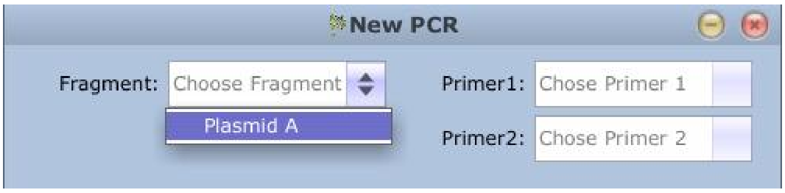

Then, select the sense primer from the Primer1 box:

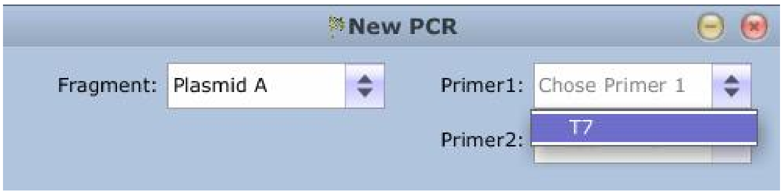

Note: only the primers annealing to the chosen fragment in a sense orientation will appear in this box.

Select the antisense primer from the Primer2 box:

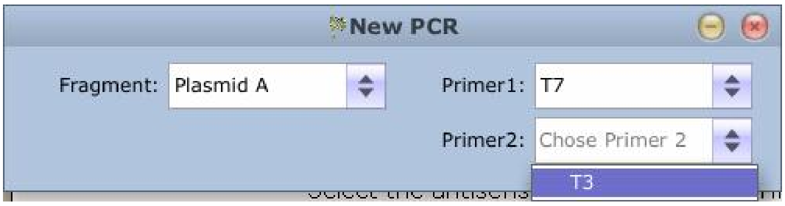

Note: once again, only the primers annealing to the chosen fragment in an antisense orientation will appear in this box.

The resulting PCR product then appears in the PCR window. To add it to your project as a new fragment, click the Add 'button. Non-annealing 5 'sequence, as added in the Add Primer window, will be automatically added on both ends of the fragment if present.

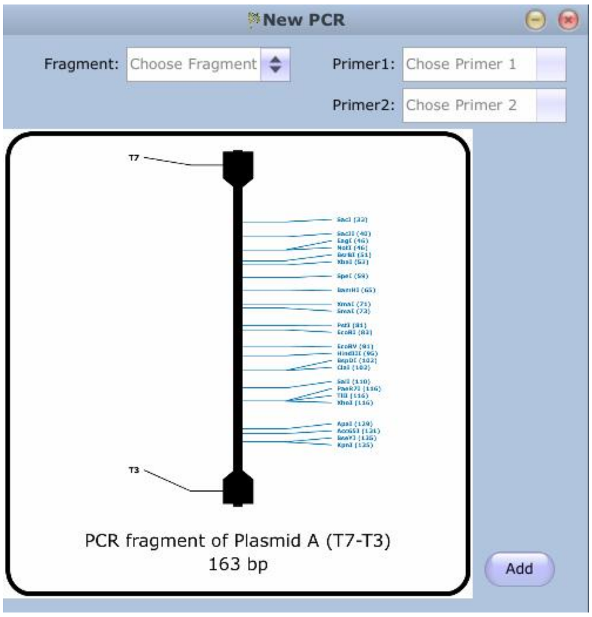

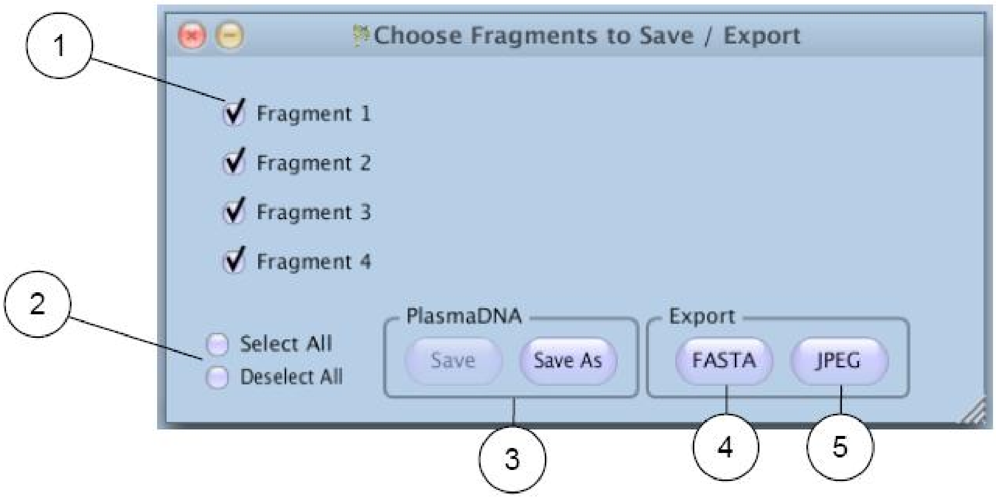

1: Select fragments to save / export. By default, all the fragments are selected.

2: Select / Deselect all fragments

3: Save fragments in a PlasmaDNA file format (.pof). This format saves the sequences, the overhangs, the selected shown enzymes/domains/primers, as well as the domains associated with the current project.

4: Export the selected sequences as a FASTA file. This format saves only the names and sequences of the fragments, but is compatible with many other programs.

5: Export the graphical view (Restriction Analysis) of one fragment in the JPEG format. If more than one fragment is selected, only the upper one will be exported.

Loading Fragments

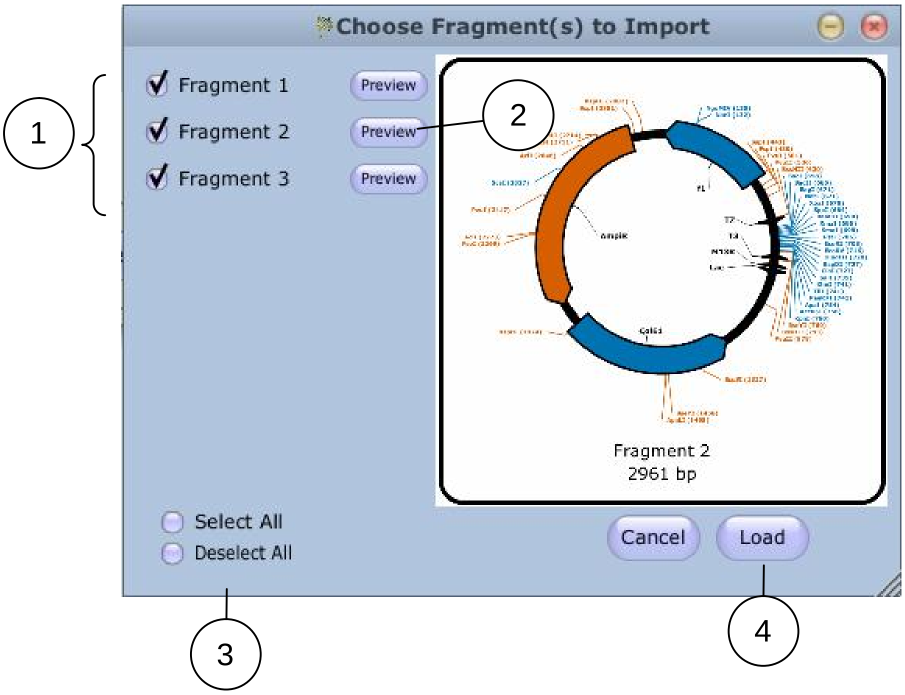

1: Fragments present in the loaded files. The selected fragments will be added to the current project.

2: Click to show a previous of this fragment.

3: Select/Deselect all fragments.

4: Add the selected fragment to the current project.

PlasmaDNA Database Manager

Introduction

Upon loading, PlasmaDNA builds a internal database of restriction enzymes, domains and primers that are used to analyse the fragments. These sequences are contained in the files Enzymes.pdat, Domains.pdat and Primers.pdat, respectively. In addition, some project files (.pof) can contain 'local" domains which are read and added to the program internal database when the savefile is loaded.

Enzymes.pdat, as supplied with the program, contains 75 restriction enzymes, corresponding to the most common non-degenerate enzymes with recognition sites of at least 6 base pairs. Domains.pdat contains 151 domains, while the Primers.pdat is empty.

Adding domains and primers to these databases is most conviently performed using PlasmaDNA as described before. However, for more advanced manipulations of these databases, it is possible to use the PlasmaDMan application supplied in the bundle.

Loading Window

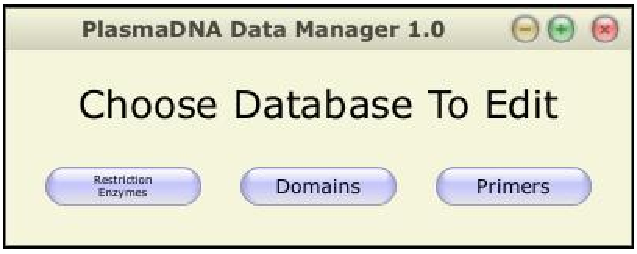

This loading window is used to select which type of database will be manipulated.

Main Window

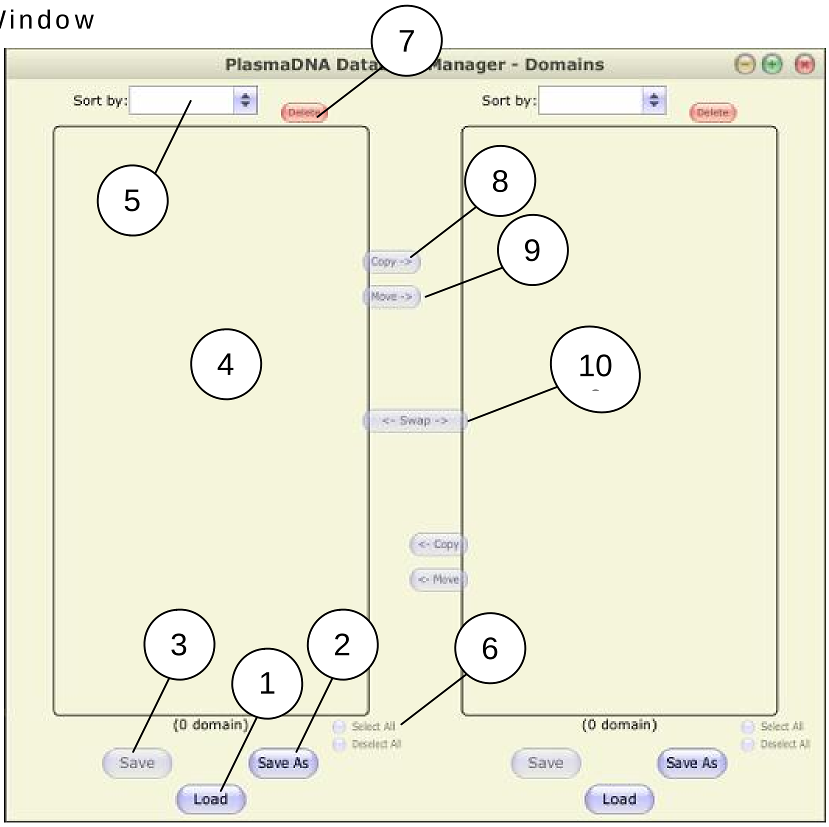

The main window is based on a "two files" concept which allows domains/enzymes/primers to be transferred from one file to the other as needed. Other possible manipulations include editing of the various fields for individual elements, as well as deleting.

1: The files are opened using the "Load" button, and saved using the "Save As" (2), or "Save" (3) buttons.

4: The different elements present in the file will appear in the window. They can be sorted according to different criteria (5), and edited directly by modifying the fields (see below for details). The different elements can be selected one by one or by using the Select All/Deselect All buttons for that file (6).

7: Delete Button, will erase the selected elements from that file
8: Copy Button, will create a copy of the selected element in the other file.
9: Move Button, will create a copy of the selected elements in the other file AND delete them from their original file
10: Swap will exchange the content of the left file with the content of the right file, and vice versa.

Note: None of the changes will be recorded until the file is saved. To cancel, just exit the program without saving.

Restriction Enzymes:

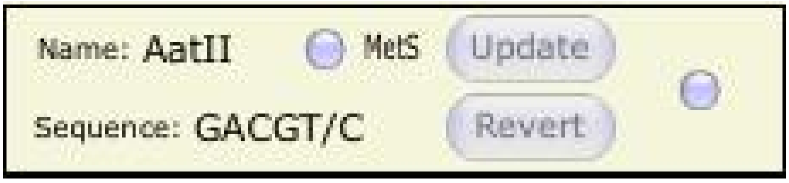

Name: Name of the Enzyme

Sequence: recongnition site of the enzyme. The cutting position is marked with the symbol "/".

MetS: Ticked if the enzyme is blocked by dam- or dcm-methylation.

Note: Restriction Enzyme database files are recognized by their name, which starts with "Enz-". When "Saving As", if the name chosen does not start with "Enz-", it will be automatically added in front.

The only database used by PlasmaDNA is the file "Enzymes.pdat" which must be found in the same folder. Other enzyme databases will not be used by PlasmaDNA but can be kept to modify the main database when needed.

To add a new enzyme to a database, fill the empty menu and click "Add".

Domains:

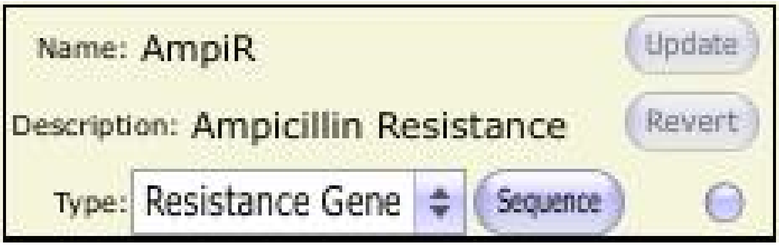

Name: Name of the domain, as it will appear on the graphical display

Description: Short description for this domain, as it will appear on the Domains info window.

Type: Type of domain. This will determine the color used in the graphical display

Sequence: Opens a subwindow to view/edit the sequence of this domain

Note: Domain database files are recognized by their name, which starts with ‘Dom-‘. When ‘Saving As”, if the name chosen does not start with ‘Dom-‘, it will be automatically added in front.

The only database used by PlasmaDNA is the file ‘Domains.pdat” which must be found in the same folder. Other domains databases will not be used by PlasmaDNA but can be kept to modify the main database when needed.

It is also possible to open project files (.pof) when editing domain databases. In this case, the domains saved in the local file (“Apply to current project”) will be displayed. Those domains can be viewed, edited, deleted and transferred as for a normal Domain database file. Saving the modifications will modify the .pof file accordingly but will not interfere with the fragments present in the project.

Unlike Restriction Enzymes and Primers, it is NOT possible to enter a new domain from scratch using Plasma DNA Domain Manager. Adding new domains must be performed using PlasmaDNA itself.

Primers:

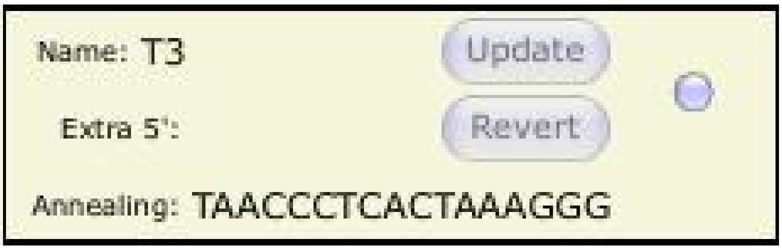

Name: Name of the primer. This field can not contain spaces, so any space present will be replaced with an underscore.

Extra: Extra, non-annealing 5’sequences found in the primer. Leave blank if none are present

Annealing: Annealing sequence of the primer.

Note: Primers database files are recognized by their name, which starts with ‘Pri-‘. When ‘Saving As”, if the name chosen does not start with ‘Pri-‘, it will be automatically added in front.

The only database used by PlasmaDNA is the file ‘Primers.pdat” which must be found in the same folder. Other primers databases will not be used by PlasmaDNA but can be kept to modify the main database when needed.

Web Resources

The PlasmaDNA website is

http://research.med.helsinki.fi/plasmadna/

The following can be be found on this site:

Download/register for updates

PC version: http://research.med.helsinki.fi/plasmadna/Download/PC_Version.htm
Mac version : http://research.med.helsinki.fi/plasmadna/Download/Mac_Version.htm

Version history

http://research.med.helsinki.fi/plasmadna/Other/Version_History.htm

File format

Savefiles : http://research.med.helsinki.fi/plasmadna/Other/SaveFile_Format.htm
Databases: http://research.med.helsinki.fi/plasmadna/Other/Database_Format.htm

Feedback

http://research.med.helsinki.fi/plasmadna/Feedback/Feedback.htm

Frequently Asked Questions

http://research.med.helsinki.fi/plasmadna/Other/FAQ.htm
