# Supplementary figures and images for: PlasmaDNA: a free, cross-platform plasmid manipulation program for molecular biology laboratories
Source: BMC Mol Biol. 2007 Sep 17;8:77. doi: 10.1186/1471-2199-8-77 (PMC2075515; doi:10.1186/1471-2199-8-77)

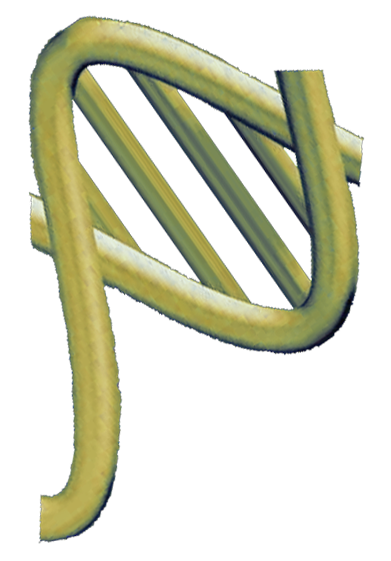

Supplement: Additional file 1 — PlasmaDNA Windows version package. Version 1.3.6 of the PlasmaDNA package for Windows [file 1471-2199-8-77-S1.zip › PlasmaDNA/PlasMa logo.png]
